# Access to cancer medicines deemed essential by oncologists in 82 countries: an international, cross-sectional survey

**DOI:** 10.1016/S1470-2045(21)00463-0

**Published:** 2021-10

**Authors:** Adam Fundytus, Manju Sengar, Dorothy Lombe, Wilma Hopman, Matthew Jalink, Bishal Gyawali, Dario Trapani, Felipe Roitberg, Elisabeth G E De Vries, Lorenzo Moja, André Ilbawi, Richard Sullivan, Christopher M Booth

**Affiliations:** a Division of Cancer Care and Epidemiology, Queen's University Cancer Research Institute, Kingston, ON, Canada; b Departments of Oncology, Queen's University, Kingston, ON, Canada; c Public Health Sciences, Queen's University, Kingston, ON, Canada; d Department of Medical Oncology, Tata Memorial Centre, Mumbai, India; e Cancer Diseases Hospital, Lusaka, Zambia; f Division of Early Drug Development for Innovative Therapies, European Institute of Oncology IRCCS, Milan, Italy; g Department of Noncommunicable Diseases, World Health Organization, Geneva, Switzerland; h Department of Health Products Policy and Standards, World Health Organization, Geneva, Switzerland; i Department of Medical Oncology, University Medical Center Groningen, University of Groningen, Groningen, Netherlands; j Institute of Cancer Policy, King's College London, London, UK

## Abstract

**Background:**

The WHO Essential Medicines List (EML) identifies priority medicines that are most important to public health. Over time, the EML has included an increasing number of cancer medicines. We aimed to investigate whether the cancer medicines in the EML are aligned with the priority medicines of frontline oncologists worldwide, and the extent to which these medicines are accessible in routine clinical practice.

**Methods:**

This international, cross-sectional survey was developed by investigators from a range of clinical practice settings across low-income to high-income countries, including members of the WHO Essential Medicines Cancer Working Group. A 28-question electronic survey was developed and disseminated to a global network of oncologists in 89 countries and regions by use of a hierarchical snowball method; each primary contact distributed the survey through their national and regional oncology associations or personal networks. The survey was open from Oct 15 to Dec 7, 2020. Fully qualified physicians who prescribe systemic anticancer therapy to adults were eligible to participate in the survey. The primary question asked respondents to select the ten cancer medicines that would provide the greatest public health benefit to their country; subsequent questions explored availability and cost of cancer medicines. Descriptive statistics were used to compare access to medicines between low-income and lower-middle-income countries, upper-middle-income countries, and high-income countries.

**Findings:**

87 country-level contacts and two regional networks were invited to participate in the survey; 46 (52%) accepted the invitation and distributed the survey. 1697 respondents opened the survey link; 423 were excluded as they did not answer the primary study question and 326 were excluded because of ineligibility. 948 eligible oncologists from 82 countries completed the survey (165 [17%] in low-income and lower-middle-income countries, 165 [17%] in upper-middle-income countries, and 618 [65%] in high-income countries). The most commonly selected medicines were doxorubicin (by 499 [53%] of 948 respondents), cisplatin (by 470 [50%]), paclitaxel (by 423 [45%]), pembrolizumab (by 414 [44%]), trastuzumab (by 402 [42%]), carboplatin (by 390 [41%]), and 5-fluorouracil (by 386 [41%]). Of the 20 most frequently selected high-priority cancer medicines, 19 (95%) are currently on the WHO EML; 12 (60%) were cytotoxic agents and 13 (65%) were granted US Food and Drug Administration regulatory approval before 2000. The proportion of respondents indicating universal availability of each top 20 medication was 9–54% in low-income and lower-middle-income countries, 13–90% in upper-middle-income countries, and 68–94% in high-income countries. The risk of catastrophic expenditure (spending >40% of total consumption net of spending on food) was more common in low-income and lower-middle-income countries, with 13–68% of respondents indicating a substantial risk of catastrophic expenditures for each of the top 20 medications in lower-middle-income countries versus 2–41% of respondents in upper-middle-income countries and 0–9% in high-income countries.

**Interpretation:**

These data demonstrate major barriers in access to core cancer medicines worldwide. These findings challenge the feasibility of adding additional expensive cancer medicines to the EML. There is an urgent need for global and country-level policy action to ensure patients with cancer globally have access to high priority medicines.

**Funding:**

None.

## Introduction

The WHO Essential Medicines List (EML) identifies priority medicines on the basis of a rigorous review of their benefits, harms, affordability, availability, and feasibility of delivery.^1^, ^2^ The EML serves as a valuable tool for policy makers of member states to optimise country-level selection of medicines and to ensure access to these drugs in the context of universal health coverage (UHC).^3^ The EML can guide the procurement of medicines, favouring competition among treatments with overlapping benefits, and is the basis of the WHO Prequalification Programme and the Medicines Patent Pool.^4^

**Research in context**

**Evidence before this study**

Our literature search was initiated on Sept 1, 2020, and concluded on Oct 1, 2020. We searched MEDLINE and PubMed for studies published up to Oct 1, 2020, using the keywords “essential medicines list”, “cancer”, and “access”. One author (AF) screened relevant abstracts and full-text articles to obtain background information on the topic. Additionally, we screened WHO technical documents on the Essential Medicines List (EML) selection process. Only English language articles and documents were included. We did not apply a formal screening process for included and excluded articles and documents. We identified only four directly relevant studies on the topic of access to medicines in oncology. A pre-existing body of literature describes the costs of cancer medicines (including those on the EML). There are major knowledge gaps about the extent to which cancer medicines listed on the EML are convergent with the priorities of frontline oncologists and whether these medicines are available in routine clinical care. We found one commentary questioning whether the EML is useful. We also identified four key studies that attempted to quantify access to oncology drugs for patients in lower-middle-income countries or upper-middle-income countries. A survey of medical oncologists showed that only 15% of patients in lower-middle-income countries in southeast Asia had access to index medications for colon cancer and lung cancer. Another report showed that economic hardship was incurred by a third of patients undergoing cancer treatment in lower-middle-income countries in southeast Asia. Two other studies used a similar survey tool to ours to ascertain the availability of several common oncology drugs in various different health systems. The authors showed that 32% of EML cancer medicines are available only at full cost in lower-middle-income countries and 5% are completely unavailable. These previous reports did not quantify the impact of drug prices on patient access. Our study aimed to address these knowledge gaps by using a modified tool and evaluated a larger sample of physicians across a greater number of upper-middle-income countries and lower-middle-income countries.

**Added value of this study**

To our knowledge, this is the first study to evaluate the concordance of medicines included in the WHO EML with those selected by clinicians and the availability of these medicines on the frontlines of clinical care. Our findings show that most medicines deemed essential by oncologists are conventional cytotoxic agents and are currently represented on the EML. However, most health systems are failing to ensure access to even these basic anticancer medicines. These problems are particularly acute in lower-middle-income countries and upper-middle-income countries, where a substantial proportion of patients will incur financial catastrophe even to pay for older cytotoxic medications.

**Implications of all the available evidence**

Although these data suggest strong convergence between the WHO EML and oncologists' perceptions of high-priority cancer medicines, we identified major barriers to drug access largely in lower-middle-income and upper-middle-income countries. These findings challenge the feasibility of adding additional expensive cancer medicines to the EML. There is an urgent need for global and country-level policy action to ensure patients with cancer in all countries have access to high-priority medicines.

The EML is designed to be adaptive to evolving global health needs.^1^ Over the past three decades, there has been a global shift from communicable towards non-communicable diseases, of which cancer is the second leading cause of mortality.^5^ The EML has evolved to reflect this epidemiological transition; only seven cancer medicines were listed in 1977 compared with 55 in 2019.^1^, ^6^

A guiding principle of the EML is that cost should not be a reason to exclude a medicine if it will make a substantial difference to population health.^7^ In listing effective but expensive medicines, it is hoped that policies to improve access to these medicines, including a reduction in prices, will be implemented; medicines for HIV and hepatitis C are good examples in this regard.^4^ To date, it is not clear whether this outcome has been achieved in oncology, where new medicines remain very expensive (typically >US$100 000 per course in high-income countries) irrespective of the magnitude of clinical benefit.^8^, ^9^ The current framework of rapid innovation in cancer, accompanied by high costs of targeted therapies and modest efficacy, raises important questions about which cancer medicines can be made accessible and which should be considered truly essential. The lack of consistency in prioritisation of medicines within the oncology community poses serious risks to health-care sustainability.

The WHO EML is developed together with clinical experts from diverse clinical practice settings. However, the extent to which practising oncologists concur with the selection of cancer medicines included on the EML is unknown. It is also not known to what extent essential cancer medicines are accessible on the frontlines of clinical care.^10^ To address these knowledge gaps and inform global policy, we aimed to investigate which medicines oncologists worldwide deem most essential in the treatment of cancer, whether the EML reflects these priority medicines, and the extent to which essential cancer medicines are available to patients in routine clinical care.

## Methods

### Study population

This international, cross-sectional survey was developed by investigators from a range of clinical practice settings in low-income and lower-middle-income countries, upper-middle-income countries, and high-income countries, with expertise in research methodology, health policy, global health, and clinical cancer care, and included members of the WHO Essential Medicines Cancer Working Group. Fully qualified physicians who deliver systemic anticancer therapy were eligible to participate in this survey. Physicians who treat only children were not eligible for inclusion in this survey; a parallel study was done to focus on paediatric cancer care, which has now been completed and will be published at a later date.

### Survey design and distribution

An electronic questionnaire was developed with the Qualtrics online survey platform (Provo, UT, USA). The survey was piloted and revised on the basis of feedback from the study team and ten additional oncologists from low-income, lower-middle-income, upper-middle-income, and high-income settings. The final survey consisted of 28 questions and took 8–10 min to complete (appendix pp 1–9). The survey was only available in English.

The anonymous survey captured information on demographics and clinical practice setting. The primary study question was as follows: “Imagine your government has put you in charge of selecting anticancer medicines for your country. You are only allowed to select a maximum of 10 medicines that will be available to treat all cancers in your country. Which drugs would you recommend to the government to achieve the greatest benefit for the most patients? Assume that cost (system and patient) is not an issue and that you have access to the necessary supportive care medicines, diagnostic and laboratory services.” The primary question was structured to prompt participants to prioritise medications on the basis of the magnitude of benefit, toxicity, and the absolute number of patients who might benefit. Respondents selected up to ten drugs from an expansive list of 164 cancer medications, which included all drugs approved by Health Canada as of September, 2020.^11^ The majority of these drugs were also approved by many other major licensing authorities such as the European Medicines Agency and the US Food and Drug Administration (FDA). Supportive care medications were excluded, except for prednisone and dexamethasone, given their cytotoxic properties in haematological cancers.

The second set of questions related to the ability of patients to access each selected medicine in routine clinical practice. These questions were based on previous work by the European Society of Medical Oncology (ESMO), with modifications to explicitly identify the risks of substantial and catastrophic expenditure.^12^, ^13^, ^14^ The scale included four categories: universally accessible (no substantial out-of-pocket expenses for >90% of patients), accessible with substantial out-of-pocket expenses (mixed or partial reimbursement model and not UHC), accessible with high risk of catastrophic expenditure (substantial out-of-pocket expenses for >50% of patients, with a substantial risk of catastrophic health expenditure defined as “spending that absorbs more than 40% of total consumption, net of food expenditures”), and unavailable for other reasons (eg, procurement or regulatory).

The sampling frame for our target population was a global network of oncologists derived from two sources: membership of national and regional oncology organisations, and personal networks of a single oncologist contact in countries where national organisations did not exist or were unable to distribute the survey (appendix pp 10–11). To minimise the risk of sampling bias, the preferred route for survey dissemination was via national and regional oncology organisations rather than personal networks. This survey distribution methodology has been successfully used previously in another global study of oncologist workload.^15^

The web survey was distributed via a hierarchical snowball method through a primary global network comprising oncologist contacts in 89 distinct countries and regions. The survey was open from Oct 15 to Dec 7, 2020; one reminder was sent to each country contact or regional contact. Contacts were asked to distribute the survey via email to their national or regional organisation. In African countries and India, the survey was distributed via formal email lists and informal WhatsApp message groups of oncologists. In the USA, the link was shared via the American Society of Clinical Oncology Twitter account.

### Statistical analysis

Survey responses were downloaded into, and all statistical analyses done with, IBM SPSS (version 26.0 for Windows). Participants were classified into three groups on the basis of the World Bank income status of their country of practice: low-income and lower-middle-income countries, upper-middle-income countries, and high-income countries.^16^ Responses that did not fully answer the primary survey question (listing of top ten medicines) were deemed incomplete and excluded. Missing data for demographic variables and access to medicines are identified in the table footnotes; percentages were calculated on the basis of those respondents who did provide a response. Frequency tables were derived for rank order of medications that were listed by respondents as most essential; comparative analyses between subgroups and analysis of drug availability were restricted to those medicines that were ranked in the top 20 globally and within each income group. The demographics and clinical practice settings of the three income groups were compared with the Pearson χ^2^ test or Fisher's exact test for categorical data, and one-way ANOVA with Tukey's post-hoc tests for age and years in practice. A p value less than 0·05 was used as the cutoff point for statistical significance and no additional adjustment was made for multiple comparisons.

### Role of the funding source

There was no funding source for this study.

## Results

87 country-level contacts and two regional networks (Latin American and Caribbean Society of Medical Oncology [SLACOM] and an informal network of oncologists in sub-Saharan Africa) were invited to participate; 46 (52%) accepted the invitation and distributed the survey via national medical oncology organisations (29 [63%] of 46), regional networks (two [4%] of 46), and informal personal networks (15 [33%] of 46). Overall, 1697 respondents opened the survey link; 423 were excluded as they did not answer the primary study question and 326 were excluded because of ineligibility (ie, they were trainees or did not prescribe chemotherapy). The final study cohort comprised 948 respondents from 82 countries. The median survey response rate was 6% (range 0–60; appendix pp 10–11).

Among the 948 respondents, 618 (65%) were from high-income countries, 165 (17%) from upper-middle-income countries, and 165 (17%) from low-income and lower-middle-income countries (table 1). 544 (66%) of 825 respondents were male and the mean age was 47 years (SD 10). 715 (75%) of 948 were medical oncologists and 160 (17%) were clinical oncologists. 474 (50%) respondents worked exclusively in a publicly funded health system.

**Table 1:** Demographic characteristics and clinical practice setting of respondents to global cancer WHO Essential Medicines List survey, stratified by World Bank economic classification

		**Total (n=948)**	**Low-income and lower-middle-income countries (n=165)**	**Upper-middle-income countries (n=165)**	**High-income countries (n=618)**	**p value**
**Demographics**						
Sex						
	Male	544/825 (66%)	101/136 (74%)	94/142 (66%)	349/547 (64%)	0·070
	Female	281/825 (34%)	35/136 (26%)	48/142 (34%)	198/547 (36%)	..
Mean age, years		47 (10)	44 (9)	48 (11)	47 (10)	0·0076*
Mean years in practice		14 (10)	11 (9)	15 (12)	14 (10)	0·0002†
**Clinical practice setting**						
Specialty						
	Medical oncology	714 (75%)	91 (55%)	110 (67%)	514 (83%)	<0·0001
	Radiation oncology	26 (3%)	17 (10%)	2 (1%)	7 (1%)	..
	Clinical oncology	159 (17%)	46 (28%)	51 (31%)	63 (10%)	..
	Other‡	49 (5%)	11 (7%)	2 (1%)	34 (6%)	..
Health system						
	Public	474/828 (57%)	51/137 (37%)	38/144 (26%)	385/547 (70%)	<0·0001
	Private	208/828 (25%)	51/137 (37%)	59/144 (41%)	98/547 (18%)	..
	Both	146/828 (18%)	35/137 (26%)	47/144 (33%)	64/547 (12%)	..
Location						
	Urban	731/828 (88%)	117/137 (85%)	133/144 (92%)	481/547 (88%)	0·15
	Rural	33/828 (4%)	9/137 (7%)	1/144 (1%)	23/547 (4%)	..
	Both	64/828 (8%)	11/137 (8%)	10/144 (7%)	43/547 (8%)	..
Type of cancer						
	Solid	612/827 (74%)	52/136 (38%)	119/144 (83%)	441/547 (81%)	<0·0001
	Haematological	55/827 (7%)	9/136 (7%)	3/144 (2%)	43/547 (8%)	..
	Both	160/827 (19%)	75/136 (55%)	22/144 (15%)	63/547 (12%)	..
Academic centre						
	Yes	580/826 (70%)	107/135 (79%)	90/144 (63%)	383/547 (70%)	0·0091
	No	246/826 (30%)	28/135 (21%)	54/144 (38%)	164/547 (30%)	..
Base of practice						
	Hospital based	714/826 (86%)	113/136 (83%)	94/144 (65%)	507/546 (93%)	<0·0001
	Clinic based	40/826 (5%)	2/136 (2%)	20/144 (14%)	18/546 (3%)	..
	Both	72/826 (9%)	21/136 (15%)	30/144 (21%)	21/546 (4%)	..
Number of cancer sites treated						
	1	218/827 (26%)	14/136 (10%)	10/144 (7%)	194/547 (36%)	<0·0001
	2	99/827 (12%)	5/136 (4%)	8/144 (6%)	86/547 (16%)	..
	≥3	510/827 (62%)	117/136 (86%)	126/144 (88%)	267/547 (49%)	..
Type of therapy						
	Systemic	803 (85%)	100 (61%)	137 (83%)	566 (92%)	<0·0001
	Both systemic and radiotherapy	145 (15%)	65 (39%)	28 (17%)	52 (8%)	..
Population treated						
	Adults only	827 (87%)	68 (41%)	155 (94%)	604 (98%)	<0·0001
	Adults and children	121 (13%)	97 (59%)	10 (6%)	14 (2%)	..

Data are n (%), n/N (%), or mean (SD). The denominator for each variable is the total number of participants as indicated in the column heading unless otherwise noted due to missing responses. Several respondents were missing much of these data since they did not complete the entire survey; percentages were calculated on the basis of those respondents who did provide a response. For low-income and lower-middle-income countries, 67 respondents were missing data for years in practice and 29 for age. For upper-middle-income countries, 60 respondents were missing data for years in practice and 23 for age. For high-income countries, 278 respondents were missing data for years in practice and 74 for age. The χ^2^ percentages are based on the subset with responses, as are the p values.

* Upper-middle-income countries and high-income countries did not differ significantly; low-income and lower-middle-income countries differed from upper-middle-income countries (p=0·0014) and from high-income countries (p=0·0002), Tukey's post-hoc test.

† Upper-middle-income countries and high-income countries did not differ significantly; low-income and lower-middle-income countries differed from upper-middle-income countries (p=0·015) and from high-income countries (p=0·012), Tukey's post-hoc test.

‡ Other specialties (n=47) included 22 haematologists, seven surgeons, three dermatologists, three gynaecologists, two gastroenterologists, one neuro-oncologist, and nine unstated.

Compared with oncologists in upper-middle-income countries and high-income countries, those from low-income and lower-middle-income countries were more likely to treat both solid and haematological tumours (75 [55%] in low-income and lower-middle-income countries, 22 [15%] in upper-middle-income countries, and 63 [12%] in high-income countries; p<0·0001); prescribe both chemotherapy and radiotherapy (65 [39%] in low-income and lower-middle-income countries, 28 [17%] in upper-middle-income countries, and 52 [8%] in high-income countries; p<0·0001), and more likely to treat adults and children (97 [59%] in low-income and lower-middle-income countries, ten [6%] in upper-middle-income countries, and 14 [2%] in high-income countries; p<0·0001). Respondents in low-income and lower-middle-income countries and upper-middle-income countries were more likely to work in the private health system than those in high-income countries (51 [37%] of 165 and 59 [41%] of 165 *vs* 98 [18%] of 618; p<0·0001).

The top 20 essential medicines selected by respondents are shown in table 2. Among these highest priority medicines, 12 (60%) of 20 were cytotoxic agents, four (20%) were targeted agents, two (10%) were immunotherapies, and two (10%) were hormonal agents. The first FDA approval was before the 1980s for six (30%) of 20 drugs, in the 1980s for one (5%), in the 1990s for six (30%), in the 2000s for four (20%), and 2010 onwards for three (15%). The most commonly selected medicines were doxorubicin, cisplatin, paclitaxel, pembrolizumab, trastuzumab, carboplatin, and 5-fluorouracil (table 2).

**Table 2:** 20 most commonly selected cancer medicines by 948 oncologists

	**Overall**		**Low-income and lower-middle-income countries**		**Upper-middle-income countries**		**High-income countries**	
	Top 20 drugs	Number of respondents (%)	Top 20 drugs	Number of respondents (%)	Top 20 drugs	Number of respondents (%)	Top 20 drugs	Number of respondents (%)
1	Doxorubicin	499 (53%)	Doxorubicin	105 (64%)	Doxorubicin	94 (57%)	Pembrolizumab*	311 (50%)
2	Cisplatin	470 (50%)	Cisplatin	91 (55%)	Pembrolizumab*	86 (52%)	Doxorubicin	300 (49%)
3	Paclitaxel	423 (45%)	Cyclophosphamide	90 (55%)	Trastuzumab	84 (51%)	Cisplatin	300 (49%)
4	Pembrolizumab	414 (44%)	Carboplatin	84 (51%)	Cisplatin	79 (48%)	5-fluorouracil	277 (45%)
5	Trastuzumab	402 (42%)	Capecitabine	80 (48%)	Carboplatin	72 (44%)	Paclitaxel	276 (45%)
6	Carboplatin	390 (41%)	Paclitaxel	79 (48%)	Paclitaxel	68 (41%)	Trastuzumab	275 (44%)
7	5-fluorouracil	386 (41%)	Docetaxel	56 (34%)	Tamoxifen	67 (41%)	Carboplatin	234 (38%)
8	Tamoxifen	345 (36%)	Tamoxifen	50 (30%)	Capecitabine	64 (39%)	Tamoxifen	228 (37%)
9	Capecitabine	329 (35%)	5-fluorouracil	49 (30%)	5-fluorouracil	60 (36%)	Capecitabine	185 (30%)
10	Cyclophosphamide	318 (34%)	Imatinib	45 (27%)	Docetaxel	57 (35%)	Oxaliplatin	184 (30%)
11	Docetaxel	296 (31%)	Gemcitabine	45 (27%)	Cyclophosphamide	51 (31%)	Docetaxel	183 (30%)
12	Oxaliplatin	269 (28%)	Trastuzumab	43 (26%)	Oxaliplatin	48 (29%)	Dexamethasone	182 (29%)
13	Dexamethasone	248 (26%)	Dexamethasone	41 (25%)	Abiraterone	41 (25%)	Cyclophosphamide	177 (29%)
14	Nivolumab	205 (22%)	Methotrexate	40 (24%)	Anastrozole	31 (19%)	Nivolumab	173 (28%)
15	Rituximab	203 (21%)	Vincristine	40 (24%)	Osimertinib†	29 (18%)	Rituximab	146 (24%)
16	Imatinib	184 (19%)	Oxaliplatin	37 (22%)	Imatinib	28 (17%)	Osimertinib†	112 (18%)
17	Gemcitabine	180 (19%)	Etoposide	36 (22%)	Goserelin	27 (16%)	Imatinib	111 (18%)
18	Etoposide	170 (18%)	Rituximab	35 (21%)	Gemcitabine	26 (16%)	Letrozole*	111 (18%)
19	Osimertinib†	157 (17%)	Bortezomib	28 (17%)	Dexamethasone	25 (15%)	Gemcitabine	109 (18%)
20	Letrozole*	143 (15%)	Gefitinib	25 (15%)	Etoposide	25 (15%)	Etoposide	109 (18%)

Data are n (%). Medicines listed are those selected by oncologists in response to the primary study question. Overall results are shown for all respondents in addition to rank order lists for three different World Bank economic classifications based on respondents' country of practice.

* Valid substitution for a listed WHO Essential Medicines List (EML) medication based on identical drug class or mechanism.

† Not included on the current WHO EML.

There was substantial agreement between the lists generated by respondents from low-income and lower-middle-income countries, upper-middle-income countries, and high-income countries; 15 (75%) of 20 medications are common to all three top 20 lists. However, although the list for low-income and lower-middle-income countries does not include any immunotherapy agents and the only hormone therapy listed is tamoxifen, the lists for upper-middle-income countries and high-income countries include immunotherapies (ie, pembrolizumab and nivolumab) and newer hormonal treatments (ie, aromatase inhibitors, goserelin, and abiraterone).

There was considerable agreement between the medications identified as high priority in this study and medications currently included on the WHO EML (figure). Among the 20 highest priority medicines identified in this study, only one drug (osimertinib) is not represented on the current EML. As shown in the figure, the medications most frequently identified as high priority by clinicians are more likely to be included on the EML. All drugs on the top 20 list for low-income and lower-middle-income countries are represented on the WHO EML. Of the bottom 100 drugs (selected by 0–2% of respondents), 16 (16%) were on the EML (appendix pp 12–16).

**Figure:**
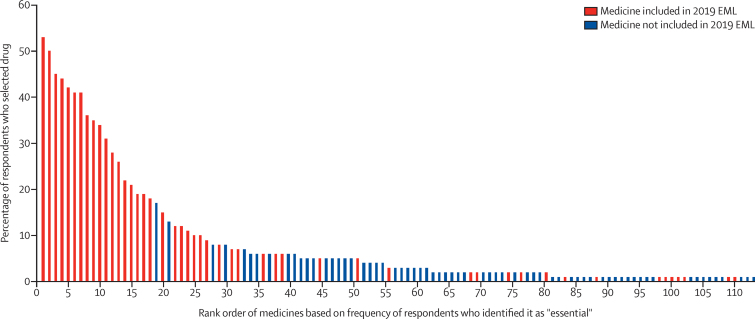
Association between rank order of all medicines identified by 948 oncologists globally as being most essential and whether the drug is listed on the 21st WHO Essential Medicines List (2019)^1^, ^2^ Only medications that received at least 1% of the vote are included in the figure. The complete rank order list with the names of the medications is available in the appendix (pp 12–16).

Reported access to the top 20 medicines varied substantially across economic settings (table 3). Among the top 20 medicines selected by oncologists in low-income and lower-middle-income countries, between 13% and 68% of respondents indicated that accessing each of these drugs placed patients at risk of catastrophic expenditure. This was the case even for older generic cytotoxic drugs such as doxorubicin and cisplatin, with 27% of oncologists reporting a substantial risk of catastrophic financial expenditures for doxorubicin and 21% for cisplatin. Of the top 20 medicines listed for low-income lower-middle-income countries, dexamethasone was the only medication that was universally available for more than 50% of respondents.

**Table 3:** Access to the 20 most frequently selected essential medicines identified by 948 oncologists, stratified by World Bank economic classification

	Overall number of responses*	**Universally available**†	**Substantial OOP expenses**‡	**Risk of catastrophic expenditure**§	**Not available**
**Top 20 medications in low-income and lower-middle-income countries**					
Doxorubicin	102	37 (36%)	33 (32%)	27 (27%)	5 (5%)
Cisplatin	77	42 (48%)	25 (28%)	18 (21%)	3 (3%)
Cyclophosphamide	88	37 (42%)	27 (31%)	20 (23%)	4 (5%)
Carboplatin	86	26 (33%)	27 (34%)	26 (33%)	0
Capecitabine	74	18 (24%)	27 (37%)	26 (35%)	3 (4%)
Paclitaxel	73	18 (25%)	31 (43%)	21 (29%)	3 (4%)
Docetaxel	55	13 (24%)	21 (38%)	19 (35%)	2 (4%)
Tamoxifen	47	17 (36%)	18 (38%)	9 (19%)	3 (6%)
5-fluorouracil	47	21 (45%)	10 (21%)	12 (26%)	4 (8%)
Imatinib	42	15 (36%)	21 (50%)	6 (14%)	0
Gemcitabine	42	8 (19%)	16 (38%)	16 (38%)	2 (5%)
Trastuzumab	41	6 (15%)	6 (15%)	28 (68%)	1 (2%)
Dexamethasone	41	22 (54%)	12 (29%)	6 (15%)	1 (2%)
Methotrexate	37	16 (43%)	12 (32%)	6 (16%)	3 (8%)
Vincristine	39	19 (49%)	7 (18%)	9 (23%)	4 (10%)
Oxaliplatin	35	8 (23%)	12 (34%)	14 (40%)	1 (3%)
Etoposide	34	13 (38%)	9 (27%)	9 (27%)	3 (9%)
Rituximab	35	3 (9%)	9 (26%)	22 (63%)	1 (3%)
Bortezomib	28	6 (21%)	12 (43%)	7 (25%)	3 (11%)
Gefitinib	24	8 (33%)	12 (50%)	3 (13%)	1 (4%)
**Top 20 medications in upper-middle-income countries**					
Doxorubicin	88	77 (88%)	5 (6%)	2 (2%)	4 (4%)
Pembrolizumab	80	10 (13%)	22 (28%)	32 (40%)	16 (20%)
Trastuzumab	79	50 (63%)	18 (23%)	7 (9%)	4 (6%)
Cisplatin	74	65 (88%)	6 (8%)	2 (3%)	1 (1%)
Carboplatin	66	55 (83%)	6 (9%)	2 (3%)	3 (5%)
Paclitaxel	64	55 (86%)	6 (9%)	2 (3%)	1 (2%)
Tamoxifen	63	54 (86%)	7 (11%)	0	2 (4%)
Capecitabine	61	45 (74%)	12 (20%)	1 (2%)	3 (5%)
5-fluorouracil	56	50 (89%)	6 (11%)	0	0
Docetaxel	51	39 (77%)	7 (14%)	2 (4%)	3 (6%)
Cyclophosphamide	48	43 (90%)	3 (6%)	1 (2%)	1 (2%)
Oxaliplatin	45	38 (84%)	6 (13%)	0	1 (2%)
Abiraterone	40	17 (43%)	11 (28%)	8 (20%)	3 (8%)
Anastrozole	30	24 (80%)	4 (13%)	0	2 (6%)
Osimertinib	27	6 (22%)	9 (33%)	11 (41%)	1 (4%)
Imatinib	27	18 (67%)	8 (30%)	1 (4%)	0
Goserelin	24	17 (71%)	6 (25%)	1 (4%)	0
Gemcitabine	24	16 (67%)	5 (21%)	2 (8%)	0
Dexamethasone	23	20 (87%)	2 (9%)	1 (4%)	0
Etoposide	24	18 (75%)	4 (17%)	0	1 (4%)
**Top 20 medications in high-income countries**					
Pembrolizumab	299	206 (69%)	62 (21%)	26 (9%)	5 (2%)
Doxorubicin	291	255 (88%)	32 (11%)	4 (1%)	0
Cisplatin	285	258 (91%)	24 (8%)	2 (1%)	1
5-fluorouracil	267	242 (91%)	25 (9%)	0	0
Paclitaxel	268	234 (87%)	31 (12%)	3 (1%)	0
Trastuzumab	263	226 (86%)	30 (11%)	5 (2%)	2 (1%)
Carboplatin	221	196 (89%)	23 (10%)	2 (1%)	0
Tamoxifen	224	197 (88%)	24 (11%)	3 (1%)	0
Capecitabine	178	142 (89%)	35 (20%)	1 (1%)	0
Oxaliplatin	180	159 (88%)	19 (11%)	2 (1%)	0
Docetaxel	175	150 (86%)	23 (13%)	2 (1%)	0
Dexamethasone	173	162 (94%)	8 (5%)	2 (1%)	1 (1%)
Cyclophosphamide	172	151 (88%)	19 (11%)	2 (1%)	0
Nivolumab	160	118 (74%)	26 (16%)	13 (8%)	3 (2%)
Rituximab	138	107 (78%)	27 (20%)	4 (3%)	0
Osimertinib	109	74 (68%)	28 (26%)	5 (5%)	2 (2%)
Imatinib	108	77 (71%)	26 (24%)	3 (3%)	2 (2%)
Letrozole	106	92 (87%)	14 (13%)	0	0
Gemcitabine	105	88 (84%)	16 (15%)	1 (1%)	0
Etoposide	105	99 (94%)	5 (5%)	0	1 (1%)

Data are n or n (%). OOP=out of pocket.

* Responses do not equal the number of respondents who selected the drug, since many respondents made their drug selections and exited the survey; the number of respondents is provided in this column.

† Available for all patients with no substantial out-of-pocket expenses for more than 90% of patients (ie, universal health-care coverage).

‡ Available for all patients with substantial out-of-pocket expenses for some patients, based on the health insurance schemes (mixed model, not universal health-care coverage).

§ Not universal health-care coverage, substantial risk of catastrophic health expenditure. Catastrophic expenditure defined as expenditure that absorbs more than 40% of total consumption net of an allowance for food expenditures.

Access to the top 20 medicines in upper-middle-income countries was higher, with more than half of respondents indicating universal availability of all medicines except for abiraterone (43% of respondents), osimertinib (22%), and pembrolizumab (13%). The number of respondents indicating that drug access was associated with a risk of catastrophic expenditure was less than 10% for all of the top 20 essential medications, except for osimertinib (41%), pembrolizumab (40%), and abiraterone (20%). However, a considerable proportion of oncologists reported a substantial risk of out-of-pocket expenditures and financial catastrophe for drugs approved in the late 1990s to early 2000s, including imatinib (30% reported a substantial risk of out-of-pocket expenditures and 4% reported a substantial risk of financial catastrophe), trastuzumab (23% and 9%), and gemcitabine (21% and 8%).

Within high-income countries, the top 20 essential cancer drugs were generally more accessible; all top 20 drugs were reported as being universally accessible by more than 65% of respondents. Across all drugs, less than 10% of respondents cited concerns about catastrophic expenditures. However, the risk of substantial out-of-pocket expenses is still present in high-income countries, as cited by 26% of respondents for osimertinib and by 24% for imatinib. Notably, even older cytotoxic drugs such as doxorubicin and paclitaxel were reported as being only accessible with substantial out-of-pocket expenses (by 11% of respondents for doxorubicin and by 12% for paclitaxel).

## Discussion

In this study, we explored which cancer medicines are deemed most essential by oncologists worldwide, the extent to which the responses of these oncologists align with the WHO EML, and the accessibility of these medicines in routine clinical practice. Several important findings have emerged from this survey. First, with only one exception (osimertinib), all of the top 20 high-priority medicines identified by clinicians globally are included on the WHO EML. Second, 70% of the top 20 medicines are older cytotoxic or hormonal agents rather than modern targeted agents or immunotherapy; 65% of the medicines received FDA regulatory approval before 2000. Third, there is high concordance (75%) among oncologists from countries of different income levels about what medicines are considered high priority. Finally, our data show striking financial barriers to accessing medicines in lower-middle-income countries; this problem even applies to older, generic, and notionally cheap cytotoxic agents. Although a greater proportion of high-priority cancer medicines are universally accessible in upper-middle-income countries and high-income countries, the risk of substantial out-of-pocket expenditures is not negligible. These results collectively suggest that although the WHO EML is generally an accurate reflection of which medicines matter most to clinicians globally, these medicines might not be sufficiently prioritised by country-level access policies, resulting in limited access to even the most fundamental regimens for basic cancer care. Notably, in almost all cases, access seems to be limited by household affordability of medicines rather than their availability.

Through the survey design, by limiting the selection to only ten medicines to treat all cancers in a country, we were able to elicit a value judgment about available therapeutics. It is encouraging to see that 19 (95%) of the top 20 highest priority medications are included in the EML. At the other end of the scale, 16 of the 100 least frequently chosen medications are also on the EML. All-trans retinoic acid, PEG-asparaginase, thioguanine, and ifosfamide might have been ranked lower by clinicians as they are used in curative regimens of rare diseases or in advanced disease where alternative options exist, or both (appendix pp 12–16). Other low-priority medicines included in the EML were nilotinib and vinorelbine (appendix pp 12–16); we speculate this is due to the availability of other treatment options in most settings. The low ranking of dacarbazine is a consequence of asking oncologists to choose only ten medicines, as dacarbazine is an important component of the ABVD (doxorubicin, bleomycin, vinblastine, and dacarbazine) treatment regimen for Hodgkin lymphoma (although alternatives do exist).

The finding that the highest priority medicines are dominated by older cytotoxic and hormonal agents challenges the common narrative of continuous new molecular breakthrough therapies. Our findings are consistent with the observation that many of these new cancer medicines are associated with marginal benefits and are lower in priority in most health systems.^8^, ^17^, ^18^ With the exception of gefitinib, all the medicines included on the top 20 lists are either core components of curative-intent regimens or have high-level evidence showing substantial gains in survival as single agents. This observation suggests that clinicians expect high-priority cancer medicines to offer meaningful gains in survival. This view has important implications for policy makers and clinical trialists, given that randomised controlled trials have largely shifted from overall survival and quality of life to endpoints such as progression-free survival.^19^, ^20^, ^21^ Three medicines in the top 30 ranking—bevacizumab, palbociclib, and epirubicin—are not currently included in the EML (appendix pp 12–16). Bevacizumab is widely used in colorectal cancer but has marginal benefit,^22^, ^23^ palbociclib has not yet shown improved survival in breast cancer,^24^, ^25^ and epirubicin can be replaced by doxorubicin in many settings. In 2016, Shulman and colleagues^26^ reported the results of a broad global consultation that identified priority medicines for the most common cancers; their results had remarkable convergence with ours. 17 of the top 20 medicines selected in our global study were also included in the list proposed by Shulman and colleagues, and two of the remaining three drugs had not yet received FDA approval.

Our data show striking barriers to accessing high-priority cancer medicines in low-income and lower-middle-income countries. Many of these core medicines were added to the EML during an extensive update in 2015.^26^ It is concerning that 5 years later, while the EML continues to add new and expensive medicines, major barriers to accessing these older core medicines remain. Less than 50% of our respondents reported universal access to generic cytotoxic drugs; core medicines such as doxorubicin, cisplatin, and tamoxifen are still associated with substantial risks of catastrophic out-of-pocket expenditures. The high rates of catastrophic expenditures for cancer medicines are consistent with previous observations that 50–90% of drug costs are borne directly by patients in resource-constrained settings.^26^ Our study reinforces the work by Cherny and colleagues,^12^ who found that 32% of essential cancer medications in lower-middle-income countries and 58% in low-income countries were available only at full price. Ultimately, the fact that a substantial proportion of patients worldwide cannot afford even older generic cytotoxic drugs with established benefits highlights a major problem with the global cancer pharmaceutical policy. As many of our respondents from upper-middle-income countries and low-income and lower-middle-income countries were from urban academic centres, it is possible that our results underestimate the problems associated with access to medicines in low-resource settings. Finally, although most of the medicines are almost certainly unaffordable in low-income and lower-middle-income countries, there are growing concerns that they are not even affordable in the health systems of upper-middle-income countries and even selected high-income countries.^27^

To our knowledge, this is the first study to evaluate the concordance of medicines included in the WHO EML with those selected by clinicians and the availability of these medicines on the frontlines of clinical care. Our findings must be considered in light of several limitations. First, the snowball methodology made it difficult to ascertain response rates in many countries but overall response was low; moreover, the survey was only administered in English. Despite substantial efforts by the study team, we had no responses from China, and respondents from low-income and lower-middle-income countries constituted only 17% of the overall cohort. Although two-thirds of the cohort was from high-income countries, one-third was collectively from upper-middle-income countries and from low-income and lower-middle-income countries, where the EML is likely to be most relevant. Although these lower numbers are a limitation, the internal consistency in the top 20 highest priority medicines across all three income groups (15 of 20 drugs are included in all three lists) is reassuring. Moreover, this study was not meant to be a definitive measure of the availability of medicines in low-income and lower-middle-income countries and upper-middle-income countries; the objective was to identify which drugs frontline clinicians value and the extent to which access to these drugs is a problem. Our data have answered these questions at a broad level; we hope this report will stimulate more granular work in the future to understand barriers to, and enablers of, priority cancer medicines at a country level. Our survey was based on a pragmatic modification of a previous survey tool rather than a validated instrument.^12^ Because our conclusions on access to medicines within a country are based on the perception of oncologists rather than actual utilisation data, there is a potential that these estimates are inaccurate. Although limiting the number of selected medicines was necessary to encourage priority setting, we recognise that restricting the list to ten medicines was an arbitrary condition that might restrict interpretation of the results. Finally, we grouped our results by World Bank income status. However, there is a large degree of heterogeneity in the health systems of low-income and lower-middle-income countries. Future work will explore medicine accessibility within specific WHO regions.

Our findings have important implications for cancer drug policies and universal health coverage. Medicines deemed essential by WHO are more likely to be included on national formularies than those that are not, even in low-income settings;^12^ these data suggest that medicines on the EML are considered important by frontline health-care providers. However, the selection of essential medicines is only one component of improving cancer care; access to these drugs is also necessary to improve population outcomes.^28^ Despite the majority of high-priority medicines being older and generic pharmaceuticals, there are major barriers to their access in low-income and lower-middle-income countries, where only a minority of oncologists reported universal accessibility. Moreover, reported risks of catastrophic expenditures were striking. Listing of a drug on a national formulary or on the EML will not ensure patient access unless the drug is eligible for reimbursement or affordable for patients, or both. Many instruments available to national authorities can increase the availability and affordability of medicines.^29^ The oncology community could learn from the HIV community, in which drug prices were markedly reduced through a multi-pronged approach that included strong UN and WHO leadership, patient advocacy, creation of global funds, voluntary licensing, and the use of patent pools.^30^ Despite a plurality of tools, there is little evidence that these tools are being used. This results in fragmented procurement and pricing systems with little power to negotiate.^31^ Countries could consider pooled procurement to leverage the power of volume, the development of biosimilar programmes, the Medicines Patent Pool to help negotiate with patent holders, and compulsory licensing. Generics and biosimilars offer the potential for substantial cost savings in all systems, including high-income countries.^32^, ^33^ Crucially, many systems lack the basic mechanisms to set priorities and to negotiate fair prices, such as health technology assessments.^34^ It is important to recognise that although prices and costs remain major barriers, these issues need to be situated within a wider context. Fragmented markets, failed tenders, inconsistent volumes, lack of reliable manufacturers (especially for generic cancer medicines), fragile supply chains and rent-seeking, mark-ups, and intermediaries extracting profit without adding value all add to the broad challenges associated with access to quality-assured cancer health products.^35^ Knowledge translation efforts are needed to identify context-specific barriers to implementation of national cancer guidelines.

Although the WHO EML reflects what the global clinical community considers essential cancer medicines, the failure to translate this knowledge into affordable and equitable access to medicines is leading to considerable avoidable suffering and mortality. It is unacceptable that high-priority medicines cannot be delivered to all patients in all health systems. There remains general consensus among oncologists worldwide on the highest-priority cancer medicines. In principle, this means health-care providers in all settings still have a similar understanding of how cancer should be treated. However, these data show that clinical practice divides are emerging; immunotherapy and other targeted agents are seen as high-priority therapeutic tools by only a small cohort of oncologists. For oncologists, a lack of consensus on what constitutes essential cancer treatment will prohibit shared learning and exchange of best practices, preclude opportunities for shared innovation and clinical trials, and further disrupt professional networks and global cohesion. Oncologists from countries across all income levels must come together in solidarity to demand integrated solutions, driven by political commitment and pragmatism, to define the highest priority interventions and to make all essential medicines for cancer available for all patients, everywhere.

## Data sharing

Survey response data for this study are not available for sharing.

## Declaration of interests

FR, LM, and AI are employees of WHO. MS, DL, LM, EGEDV, DT, FR, RS, BG, and CMB are members of the WHO Essential Medicines List Cancer Medicine Working Group. EGEDV reports institutional financial support for research from Amgen, Bayer, Crescendo Biologics, CytomX Therapeutics, G1 Therapeutics, Genentech, Regeneron, Roche, Servier, and Synthon, and institutional financial support for participation on data safety monitoring boards and advisory boards of Daiichi Sankyo, NSABP, and Crescendo Biologics. FR reports honoraria for lectures from Dr Reddy's and payments for participation on advisory boards from Boehringer-Ingelheim and MSD. BG reports consulting fees from Vivio Health. All other authors declare no competing interests.
