# Supplementary material for: Access to cancer medicines deemed essential by oncologists in 82 countries: an international, cross-sectional survey
Source: Lancet Oncol. 2021 Oct;22(10):1367–77. doi: 10.1016/S1470-2045(21)00463-0 (PMC8476341; doi:10.1016/S1470-2045(21)00463-0)
Supplement: Supplementary appendix [file mmc1.pdf]

THE LANCET
Oncology

Supplementary appendix

This appendix formed part of the original submission and has been peer reviewed. We post it as supplied by the authors.

Supplement to: Fundytus A, Sengar M, Lombe D, et al. Access to cancer medicines deemed essential by oncologists in 82 countries: an international, cross-sectional survey. *Lancet Oncol* 2021; published online Sept 21. http://dx.doi.org/10.1016/S1470-2045(21)00463-0.

Supplementary Appendix 1

Survey Questionnaire and Possible Responses to WHO EML Survey

Thank you for agreeing to participate in this WHO project. The objectives of this study are to understand which cancer medicines front-line clinicians consider essential to cancer care, and the extent to which these medicines are available in routine practice.

Survey Questionnaire

1a. Which country do you currently practice in?
A 218-country dropdown list derived from the world bank was available for selection.

1b. Other Country (Free Text)

2. Are you a:

- ○ Medical Oncologist that prescribes systemic anti-cancer therapy
- ○ Radiation Oncologist that prescribes systemic anti-cancer therapy
- ○ Clinical Oncologist that prescribes systemic anti-cancer therapy
- ○ Other Physician that prescribes systemic anti-cancer therapy
- ○ I do not prescribe systemic anti-cancer therapy

3. Are you currently an oncology trainee (Medical Student, Resident or Fellow)?

- ○ Yes
- ○ No

4. Do you provide:

- ○ Systemic anti-cancer therapy
- ○ Radiation therapy only
- ○ Both systemic anti-cancer therapy and radiation therapy
- ○ Neither systemic anti-cancer therapy nor radiation therapy

5. Do you administer cancer treatment to children?

- ○ No
- ○ Yes but I treat adults as well
- ○ Yes I treat exclusively children

6. A dedicated survey for pediatric oncologists is being distributed through the International Society of Pediatric Oncology (SIOP).

If you are not a SIOP member and still wish to participate, please contact Christopher Booth: christopher.booth@kingstonhsc.ca

7. Imagine your government has put you in charge of selecting anti-cancer medicines for the country. You are only allowed to select a maximum of 10 medicines that will be available to treat
all cancers in your country. Which drugs would you recommend to the government to achieve the greatest benefit for the most patients? Assume that cost (system and patient) is not an issue
and that you have access to the necessary supportive care medicines, diagnostic, and laboratory services).

- ▢ Abemaciclib
- ▢ Abiraterone
- ▢ Acalabrutinib
- ▢ Afatinib
- ▢ Aldesleukin
- ▢ Alectinib
- ▢ Alemtuzumab
- ▢ Amsacrine
- ▢ Anagrelide
- ▢ Anastrozole
- ▢ Apalutamide
- ▢ Arsenic Trioxide
- ▢ Atezolizumab
- ▢ Avelumab
- ▢ Axitinib
- ▢ Azacitidine
- ▢ Bendamustine
- ▢ Bevacizumab
- ▢ Bicalutamide
- ▢ Bleomycin
- ▢ Blinatumomab
- ▢ Bortezomib
- ▢ Bosutinib
- ▢ Brentuximab Vedotin
- ▢ Brigatinib
- ▢ Buserelin
- ▢ Busulfan
- ▢ Cabazitaxel
- ▢ Cabozantinib
- ▢ Capecitabine
- ▢ Carboplatin
- ▢ Carfilzomib

- ▢ Carmustine
- ▢ Ceritinib
- ▢ Cetuximab
- ▢ Chlorambucil
- ▢ Cisplatin
- ▢ Cladribine
- ▢ Cobimetinib
- ▢ Crizotinib
- ▢ Cyclophosphamide
- ▢ Cytarabine
- ▢ Dabrafenib
- ▢ Dacarbazine
- ▢ Dactinomycin
- ▢ Daratumumab
- ▢ Dasatanib
- ▢ Danorubicin
- ▢ Decitabine
- ▢ Degarelix
- ▢ Denosumab
- ▢ Dexamethasone
- ▢ Dinutuximab
- ▢ Docetaxel
- ▢ Doxorubicin
- ▢ Durvalumab
- ▢ Elotuzumab
- ▢ Enzalutamide
- ▢ Epirubicin
- ▢ Eribulin
- ▢ Erlotinib
- ▢ Erwinia Asparaginase
- ▢ Etoposide
- ▢ Everolimus
- ▢ Exemestane
- ▢ Fludarabine
- ▢ 5-Fluorouracil
- ▢ Flutamide
- ▢ Fulvestrant
- ▢ Gefitinib
- ▢ Gemcitabine
- ▢ Goserelin
- ▢ Hydroxyurea
- ▢ Ibrutinib
- ▢ Idarubicin
- ▢ Idelalisib

- ▢ Ifosfamide
- ▢ Imatinib
- ▢ Inotuzumab ozogamicin
- ▢ Interferon alfa-2b
- ▢ Ipilimumab
- ▢ Irinotecan
- ▢ Ixazomib
- ▢ Trastuzumab Emtansine (TDM-1)
- ▢ L-Asparaginase
- ▢ Lanreotide
- ▢ Lapatinib
- ▢ Lenalidomide
- ▢ Lenvatinib
- ▢ Letrozole
- ▢ Leucovorin
- ▢ Leuprolide
- ▢ Liposomal Irinotecan
- ▢ Lomustine
- ▢ Lorlatinib
- ▢ Medroxyprogesterone
- ▢ Megestrol Acetate
- ▢ Melphalan
- ▢ Mercaptopurine
- ▢ Methotrexate
- ▢ Midostaurin
- ▢ Mitomycin
- ▢ Mitotane
- ▢ Mitoxantrone
- ▢ Nab-Paclitaxel
- ▢ Nelarabine
- ▢ Nilotinib
- ▢ Nilutamide
- ▢ Nivolumab
- ▢ Obinutuzumab
- ▢ Octreotide
- ▢ Olaparib
- ▢ Osimertinib
- ▢ Oxaliplatin
- ▢ Paclitaxel
- ▢ Palbociclib
- ▢ Pamidronate
- ▢ Panitumumab
- ▢ Pazopanib
- ▢ Pegaspargase

- ▢ Pegylated liposomal Doxorubicin
- ▢ Pembrolizumab
- ▢ Pemetrexed
- ▢ Pertuzumab
- ▢ Pomalidomide
- ▢ Ponatinib
- ▢ Pralatrexate
- ▢ Prednisone
- ▢ Procarbazine
- ▢ Radium-223
- ▢ Raltitrexed
- ▢ Ramucirumab
- ▢ Regorafenib
- ▢ Ribociclib
- ▢ Rituximab
- ▢ Romidepsin
- ▢ Ruxolitinib
- ▢ Siltuximab
- ▢ Sorafenib
- ▢ Strontium-89
- ▢ Sunitinib
- ▢ Tamoxifen
- ▢ Talazoparib
- ▢ Temozolomide
- ▢ Temsirolimus
- ▢ Thalidomide
- ▢ Thioguanine
- ▢ Topotecan
- ▢ Trabectedin
- ▢ Trametinib
- ▢ Trastuzumab
- ▢ Tretinoin (ATRA)
- ▢ Trifluridine / Tipiracil
- ▢ Triptorelin
- ▢ Vandetanib
- ▢ Veleparib
- ▢ Vemurafinib
- ▢ Venetoclax
- ▢ Vinblastine
- ▢ Vincristine
- ▢ Vinorelbine
- ▢ Vismodegib
- ▢ Vorinostat
- ▢ Zoledronic acid

▢ Other ____

8. What is the current access to each of Drug 1-10 selected by participant in question 7 (was auto populated using Qualtrics code) in your practice setting?

- ○ Available for all patients with no significant out of pocket expenses for more than 90% of patients (i.e. universal healthcare coverage)
- ○ Available for all patients with significant out of pocket expenses for some patients, based on the health insurance schemes (mixed model, not universal healthcare coverage)
- ○ Available for all the patients with significant out of pocket expenses for more than half of the patients (not universal healthcare coverage, substantial risk of catastrophic health expenditure*) * spending that absorbs more than 40% of total consumption, net of an allowance for food expenditures
- ○ Not readily available due to problems with drug supply/procurement/production
- ○ Not available as drug is not approved in my country
- ○ Not available for other reasons (Free text provided if selected)

9. Below is the list of medicines you previously selected for your country assuming cost was NOT an issue. If your government now tells you that drug cost IS an issue - would you remove any of these drugs from the list? (Participants 10 selected drugs are displayed with checkbox)

10. Below is a list of cancer medicines (not including supportive care) currently listed on the WHO EML. [c] denotes drugs present on the children's EML.

11. Are there important cancer medications which are not currently listed that should be considered for addition to the WHO EML?

- ○ Yes
- ○ No

12. Please list the important cancer medications that are not currently listed that should be considered. (Free Text Box Available)

WHO EML

Cytotoxic Medicines
Arsenic Trioxide [c]
Asparaginase [c]
Bendamustine
Bleomycin [c]
Calcium Folinate [c]
Capecitabine
Carboplatin [c]
Chlorambucil
Cisplatin [c]
Cyclophosphamide [c]
Cytarabine [c]
Dacarbazine [c]
Dactinomycin [c]
Daunorubicin [c]
Docetaxel
Doxorubicin [c]
Etoposide [c]
Fludarabine
Fluorouracil [c]
Gemcitabine
Hydroxycarbamide [c]
Ifosfamide [c]
Irinotecan [c]
Melphalan
Mercaptopurine [c]
Methotrexate [c]
Oxaliplatin [c]
Paclitaxel [c]
Pegaspargase [c]
Procarbazine [c]
Realgar-Indigo naturalis formulation[c]
Tioguanine [c]
Vinblastine [c]
Vincristine [c]
Vinorelbine

Targeted Therapies
All-trans retinoid acid (ATRA) [c]
Bortezomib
Dastinib [c]
Erlotinib
Imatinib [c]
Nilotinib [c]
Rituximab [c]
Trastuzumab

Immunomodulators
Filgrastim [c]
Lenalidomide
Nivolumab
Thalidomide

Hormones and Antihormones
Abiraterone
Anastrozole
Bicalutamide
Dexamethasone [c]
Hydrocortisone [c]
Leuprorelin
Methylprednisolone [c]
Prednisolone [c]
Tamoxifen

Supportive Medicines
Allopurinol [c]
Mesna [c]
Zoledronic Acid

13. Is there a national EML list (that includes cancer medicines) in your country?

- ○ Yes
- ○ No
- ○ I don't know

14. Is the national EML list shaped on the WHO list?

- ○ Yes
- ○ No
- ○ I don't know

15. The use of a national EML improves the access of patients to cancer treatments?

- ○ Strongly Disagree
- ○ Disagree
- ○ Neutral
- ○ Agree

- ○ Strongly Agree

16. The current WHO EML improves the access of patients to cancer treatments?

- ○ Strongly Disagree
- ○ Disagree
- ○ Neutral
- ○ Agree
- ○ Strongly Agree

17. Do you play a role in any of the following drug guideline related activities? (Check all that Apply)

- ☐ Formulary (hospital, regional, or national)
- ☐ Health Technology Assessment
- ☐ Guideline Writing
- ☐ Regulatory or Funding Decision Making

18. Do you work in the:

- ○ Public health care system
- ○ Private health care system
- ○ Both

19. Is your primary practice located in an urban or rural center? Note: rural is defined as a population of less than fifty thousand and not within a two hour reasonable commuting distance of a large center

- ○ Urban
- ○ Rural
- ○ I work in both

20. Which types of cancer do you treat?

- ○ Solid Tumors
- ○ Hematological Malignancy
- ○ Both

21. Which cancers do you treat? (Check all that apply)

- ☐ Brain
- ☐ Breast
- ☐ Gastrointestinal
- ☐ Genitourinary
- ☐ Gynecological
- ☐ Head and Neck
- ☐ Lung
- ☐ Sarcoma
- ☐ Skin/Cutaneous
- ☐ Hematologic
- ☐ Other

22. Do you currently work in an academic center?

- ○ Yes
- ○ No

23. What describes your current practice best?

- ○ Hospital-based clinic
- ○ Clinic not affiliated with hospital
- ○ Both

24. Please state the number of years of practice in oncology since you completed your training (Free text box)

25.Gender

- ○ Male
- ○ Female
- ○ Other

26. Age (Free text Box)

27. Is there anything else you would like to share regarding the WHO EML? (Free Text Box)

28a. If you would be willing to be contacted for follow-up of the study, please provide your name and email address:

- ○ Yes
- ○ No

28b. Name (Free Text Box)

28c. Email (Free Text Box)

Appendix 2- Distribution Networks and Response Rates

Country/Region	Contact Type	# of responses	Denominator	Response Rate
**North America**				
Canada	National organization	43	213	20%
United States	National organization	100	Unknown	Unknown
Caribbean	Personal network	9	Unknown	Unknown
**South America**				
Latin America	Regional network	22	Unknown	Unknown
Argentina	National organization	27	170	16%
Brazil	National organization	39	2073	2%
Chile	National organization	7	Unknown	Unknown
Venezuela	Personal network	3	5	60%
**Europe**				
Andorra	Personal network	1	2	50%
Belgium	Personal network	10	Unknown	Unknown
Denmark	National organization	1	Unknown	Unknown
Estonia	National organization	7	35	20%
Finland	National organization	8	300	3%
France	Personal network	29	243	12%
Greece	National organization	25	378	7%
Hungary	National organization	43	Unknown	Unknown
Luxembourg	National organization	1	20	5%
Netherlands	Personal network	1	Unknown	Unknown
Portugal	National organization	24	811	3%
Romania	National organization	7	300	2%
Russia	National organization	1	1350	0%
Serbia	Personal network	1	100	1%
Slovenia	National organization	13	46	28%
Spain	National organization	41	Unknown	Unknown
Switzerland	Personal network	27	Unknown	Unknown
Turkey	Personal network	30	Unknown	Unknown
UK	National organization	14	1000	1%
**Asia**				
Bangladesh	Personal network	2	123	2%

India	Personal network	84	Unknown	Unknown
Japan	National organization	141	6842	2%
Korea	National organization	13	500	3%
Malaysia	National organization	9	120	8%
Nepal	National organization	9	Unknown	Unknown
Pakistan	Personal network	21	60	35%
Singapore	National organization	9	130	7%
Sri Lanka	National organization	6	Unknown	Unknown
Taiwan	National organization	20	Unknown	Unknown
**Middle East**				
Iraq	National organization	2	Unknown	Unknown
Israel	Personal network	2	Unknown	Unknown
Jordan	National organization	7	Unknown	Unknown
Kuwait	Personal network	7	Unknown	Unknown
Lebanon	National organization	12	179	7%
UAE	National organization	4	95	4%
**Sub Saharan Africa**				
Sub Saharan Africa	Regional Network	32	Unknown	Unknown
**Oceania**				
Australia	National organization	38	Unknown	Unknown
New Zealand	Personal network	3	Unknown	Unknown

Note: 89 countries and regional networks (SLACOM, Sub-Saharan Africa) were invited to participate in this study. This table shows responses from the 46 countries/regional networks who accepted the invitation and distributed the survey. From these 46 networks, the study had 948 respondents from 82 different countries.

Appendix 3-Complete List of "Essential" Drugs Ranked by Frequency of Selection by 948 Oncologists in Global Essential Medicines Survey

Rank	Drug	Frequency of Selection	% of Oncologists who Chose Drug on List of Top 10 Most Essential
1	***Doxorubicin***	***499***	***53***
2	***Cisplatin***	***470***	***50***
3	***Paclitaxel***	***423***	***45***
4	***Pembrolizumab***	***413***	***44***
5	***Trastuzumab***	***399***	***42***
6	***Carboplatin***	***390***	***41***
7	***5-Fluorouracil***	***386***	***41***
8	***Tamoxifen***	***345***	***36***
9	***Capecitabine***	***329***	***35***
10	***Cyclophosphamide***	***318***	***34***
11	***Docetaxel***	***296***	***31***
12	***Oxaliplatin***	***269***	***28***
13	***Dexamethasone***	***248***	***26***
14	***Nivolumab***	***204***	***22***
15	***Rituximab***	***203***	***21***
16	***Imatinib***	***184***	***19***
17	***Gemcitabine***	***180***	***19***
18	***Etoposide***	***170***	***18***
19	Osimertinib	157	17
20	***Letrozole***	***143***	***15***
21	Bevacizumab	120	13
22	***Abiraterone***	***118***	***12***
23	***Goserelin^***	***110***	***12***
24	***Anastrozole***	***100***	***11***
25	***Irinotecan***	***99***	***10***
26	***Methotrexate***	***95***	***10***
27	***Vincristine***	***86***	***9***
28	Palbociclib	75	8
29	***Cytarabine***	***73***	***8***
30	Epirubicin	71	8
31	***Prednisone***	***68***	***7***

32	***Zoledronic acid***	***67***	***7***
33	Pemetrexed	63	7
34	Alectinib	61	6
35	Atezolizumab	59	6
36	***Bortezomib***	***59***	***6***
37	Cetuximab	58	6
38	***Leuprolide***	***57***	***6***
39	***Bleomycin***	***56***	***6***
40	Nab-Paclitaxel	56	6
41	Temozolomide	51	5
42	Pertuzumab	50	5
43	Sunitinib	49	5
44	***Gefitinib***	***48***	***5***
45	Ipilimumab	47	5
46	Denosumab	46	5
47	Olaparib	46	5
48	Enzalutamide	45	5
49	Trastuzumab Emtansine (TDM-1)	45	5
50	***Lenalidomide***	***44***	***5***
51	Leucovorin	42	4
52	Fulvestrant	37	4
53	Ribociclib	35	4
54	Ibrutinib	34	4
55	***Erlotinib***	***32***	***3***
56	Dasatanib	31	3
57	Afatinib	28	3
58	Cabozantinib	26	3
59	Lenvatinib	25	3
60	Durvalumab	24	3
61	Lorlatinib	24	3
62	Sorafenib	23	2
63	Brentuximab Vedotin	22	2
64	Danorubicin	22	2
65	Panitumumab	22	2
66	Daratumumab	21	2
67	Hydroxyurea	21	2
68	***Ifosfamide***	***21***	***2***

**69**	**Arsenic Trioxide**	**20**	**2**
70	Crizotinib	20	2
71	Pazopanib	19	2
72	Venetoclax	19	2
73	Azacitidine	18	2
74	***Bendamustine***	***17***	***2***
75	Dabrafenib	17	2
76	***Bicalutamide***	***16***	***2***
77	Axitinib	15	2
78	Pegylated liposomal Doxorubicin	15	2
79	Ramucirumab	15	2
80	***Tretinoin (ATRA)***	***14***	***2***
81	Degarelix	13	1
82	Apalutamide	12	1
83	***Dacarbazine***	***12***	***1***
84	Cabazitaxel	11	1
85	Other	11	1
86	Brigatinib	10	1
87	Exemestane	10	1
88	Idarubicin	9	1
89	***Melphalan***	***9***	***1***
90	Trametinib	9	1
91	Eribulin	8	1
92	Everolimus	8	1
93	Ponatinib	8	1
94	Regorafenib	8	1
95	Acalabrutinib	7	1
96	Blinatumomab	7	1
97	Octreotide	7	1
98	Pomalidomide	7	1
99	***Vinblastine***	***7***	***1***
100	***Dactinomycin***	***6***	***1***
101	***Fludarabine***	***6***	***1***
102	***Mercaptopurine***	***6***	***1***
103	***Nilotinib***	***6***	***1***
104	Pamidronate	6	1
105	Vemurafinib	6	1
106	Avelumab	5	1

107	Cobimetinib	5	1
108	Inotuzumab ozogamicin	5	1
109	***Pegaspargase***	***5***	***1***
110	***Thalidomide***	***5***	***1***
111	Topotecan	5	1
112	Triptorelin	5	1
113	Alemtuzumab	4	0
114	Carfilzomib	4	0
115	Chlorambucil	4	0
116	Decitabine	4	0
117	Flutamide	4	0
118	L-Asparaginase	4	0
119	Lanreotide	4	0
120	Medroxyprogesterone	4	0
121	Midostaurin	4	0
122	Obinutuzumab	4	0
123	Radium-223	4	0
124	Ruxolitinib	4	0
125	Talazoparib	4	0
126	Trifluridine / Tipiracil	4	0
127	Busulfan	3	0
128	Erwinia Asparaginase	3	0
129	Lomustine	3	0
130	Mitomycin	3	0
131	Nilutamide	3	0
132	Bosutinib	2	0
133	Ceritinib	2	0
134	Cladribine	2	0
135	Interferon alfa-2b	2	0
136	Lapatinib	2	0
137	Megestrol Acetate	2	0
138	Nelarabine	2	0
139	***Vinorelbine***	***2***	***0***
140	Vismodegib	2	0
141	Anagrelide	1	0
142	Buserelin	1	0
143	Dinutuximab	1	0
144	Idelalisib	1	0

145	Ixazomib	1	0
146	Liposomal Irinotecan	1	0
147	Mitotane	1	0
148	Procarbazine	1	0
149	Trabectedin	1	0
150	Veleparib	1	0
151	Aldesleukin	0	0
152	Amsacrine	0	0
153	Carmustine	0	0
154	Elotuzumab	0	0
155	Mitoxantrone	0	0
156	Pralatrexate	0	0
157	Raltitrexed	0	0
158	Romidepsin	0	0
159	Siltuximab	0	0
160	Strontium-89	0	0
161	Temsirolimus	0	0
162	***Thioguanine***	***0***	***0***
163	Vandetanib	0	0
164	Vorinostat	0	0

^Not explicitly on EML, but therapeutic interchange with leuprorelin is permitted as these medications are of the same class. Therefore it was listed as included.
Bolded entries are represented on the 21st EML (2019)
